# Abnormal blood lactate accumulation during repeated exercise testing in myalgic encephalomyelitis/chronic fatigue syndrome

**DOI:** 10.14814/phy2.14138

**Published:** 2019-06-03

**Authors:** Katarina Lien, Bjørn Johansen, Marit B. Veierød, Annicke S. Haslestad, Siv K. Bøhn, Morten N. Melsom, Kristin R. Kardel, Per O. Iversen

**Affiliations:** ^1^ Department of Nutrition Institute of Basic Medical Sciences University of Oslo Oslo Norway; ^2^ CFS/ME Centre Division of Medicine Oslo University Hospital Oslo Norway; ^3^ Department of Respiratory Diseases Rikshospitalet Oslo University Hospital Oslo Norway; ^4^ Oslo Centre for Biostatistics and Epidemiology Department of Biostatistics Institute of Basic Medical Sciences University of Oslo Oslo Norway; ^5^ Department of Pulmonary Medicine The Glittre Clinic Hakadal Norway; ^6^ Department of Hematology Oslo University Hospital Oslo Norway

**Keywords:** Elevated lactate, exercise intolerance, metabolism, oxygen uptake, post‐exertional malaise

## Abstract

Post‐exertional malaise and delayed recovery are hallmark symptoms of myalgic encephalomyelitis/chronic fatigue syndrome (ME/CFS). Studies on repeated cardiopulmonary exercise testing (CPET) show that previous exercise negatively affects oxygen uptake (VO_2_) and power output (PO) in ME/CFS. Whether this affects arterial lactate concentrations ([La_a_]) is unknown. We studied 18 female patients (18–50 years) fulfilling the Canadian Consensus Criteria for ME/CFS and 15 healthy females (18–50 years) who underwent repeated CPETs 24 h apart (CPET_1_ and CPET_2_) with [La_a_] measured every 30th second. VO_2_ at peak exercise (VO_2peak_) was lower in patients than in controls on CPET_1_ (*P* < 0.001) and decreased in patients on CPET_2_ (*P* < 0.001). However, the difference in VO_2peak_ between CPETs did not differ significantly between groups. [La_a_] per PO was higher in patients during both CPETs (*P*_interaction_ < 0.001), but increased in patients and decreased in controls from CPET_1_ to CPET_2_ (*P*_*interaction*_ < 0.001). Patients had lower VO_2_ (*P* = 0.02) and PO (*P* = 0.002) at the gas exchange threshold (GET, the point where CO_2_ production increases relative to VO_2_), but relative intensity (%VO_2peak_) and [La_a_] at GET did not differ significantly from controls on CPET_1_. Patients had a reduction in VO_2_ (*P* = 0.02) and PO (*P* = 0.01) at GET on CPET_2_, but no significant differences in %VO_2peak_ and [La_a_] at GET between CPETs. Controls had no significant differences in VO_2_, PO or %VO_2peak_ at GET between CPETs, but [La_a_] at GET was reduced on CPET_2_ (*P* = 0.008). In conclusion, previous exercise deteriorates physical performance and increases [La_a_] during exercise in patients with ME/CFS while it lowers [La_a_] in healthy subjects.

## Introduction

Myalgic Encephalomyelitis/Chronic Fatigue Syndrome (ME/CFS) is a complex, multisystem and often debilitating disorder of unknown etiology (Institute of Medicine 2015). Common symptoms include fatigue, post‐exertional malaise (PEM), widespread pain, sleep disturbances, cognitive dysfunction, sensory hypersensitivity, orthostatic intolerance, and gastrointestinal discomfort. There are no reliable diagnostic markers or clinical findings that can verify the diagnosis, which is based on self‐reported symptoms. There is no widely accepted agreement regarding diagnostic criteria. The Canadian Consensus Criteria (CCC) (Carruthers et al. 2003) and The International Consensus Criteria (ICC) (Carruthers et al. 2011) both appear to identify a subgroup of patients with more severe functional impairments, as well as marked physical and cognitive symptoms (Jason et al. 2013). PEM is defined as a substantial worsening of symptoms after mild to moderate physical, mental, or emotional exertion. PEM and impaired recovery after exertion are mandatory criteria in both the CCC and the ICC.

Cardiopulmonary exercise testing (CPET) provides an accurate and objective assessment of functional capacity (ATS/ACCP Statement on cardiopulmonary exercise testing, 2003; Arena et al. 2007). Repeated CPET have demonstrated that ME/CFS patients are unable to reproduce oxygen uptake and power output at peak exercise (VO_2peak_) and/or at the gas exchange threshold defined by the V‐slope method, when tested on two consecutive days (Vermeulen et al. 2010; Snell et al. 2013; Keller et al. 2014; Nelson et al. 2019). The gas exchange threshold is the point where VCO_2_ increases relative to VO_2,_ and has traditionally been viewed as a transition from aerobic to anaerobic energy production, coinciding with the onset of lactate accumulation. However, the notion of tissue hypoxia as the main cause of lactate accumulation is no longer accepted (Brooks 2018). Lactate is always the end result of glycolysis, also under aerobic conditions (Rogatzki et al. 2015). Glycolysis increases during exercise, and lactate is rapidly cleared from the circulation, mainly through oxidation and gluconeogenesis. During an incremental exercise test, blood lactate accumulation occurs when the rate of lactate appearance exceeds the rate of lactate disposal (Brooks 2018). Trained individuals have a lower blood lactate concentration and increased lactate metabolic clearance rate for an absolute exercise intensity, compared to untrained individuals. However, the rate of lactate appearance has been found to be similar in trained and untrained subjects at the same relative intensity defined as percent of VO_2peak_ (%VO_2peak_) (Bergman et al. 1999; Messonnier et al. 2013).

Previous studies have not demonstrated consistent results with regard to physical performance and lactate accumulation in ME/CFS (Riley et al. 1990; Wong et al. 1992; Gibson et al. 1993; Lane et al. 1998; Sargent et al. 2002; Jammes et al. 2005), but lactate accumulation during repeated exercise testing has not been examined. The aim of this study was therefore to examine if VO_2_ and arterial plasma lactate concentrations at various exercise intensities differed between patients with ME/CFS and healthy controls by performing two CPETs 24 h apart.

## Materials and Methods

### Approvals

The study protocol was approved by the Regional Committee for Medical and Health Research Ethics in Norway (no. 2012/571‐1), and is registered with ClinicalTrials.org (ID NCT02970240).

### Subjects

We used social network and media coverage to invite potential participants, and we received a large number of requests to participate in this study. All requests were pre‐screened with regard to age (18–50 years), gender (females), place of living (in the study area), health status, medication, and current level of physical activity. Eligible candidates were interviewed consecutively on telephone or in person. All included patients fulfilled the CCC for ME/CFS (Carruthers et al. 2003). We excluded patients who were pregnant, bedridden or had comorbidities that could interfere with CPET results, that is, lung‐ and heart disorders, or used medication known to affect physical performance. The controls had no known previous or current serious illness, did not use any regular medication (oral contraceptives were allowed), had no first‐grade relatives with ME/CSF and exercised less than twice weekly on a regular basis. To minimize PEM due to traveling to the study site, the patients were offered to stay at the study site during the test period. Eighteen female patients with ME/CFS and 15 female healthy controls participated in the study after signing informed consent forms.

### Exercise testing

All tests were performed between 8 and 11 am at the Glittre Clinic, a rehabilitation hospital for patients with chronic pulmonary diseases. The participants were asked to refrain from physical exertion 72 h prior to the first CPET and were tested after an overnight fasting, allowing free consumption of water. Weight (to the nearest 0.5 kg) and height (to the nearest 1.0 cm) were recorded and a spirometry was performed at baseline the first day (Oxycon Pro, Erich Jaeger GmbH, Wurzburg, Germany). Baseline electrocardiogram (ECG) was monitored on both days (CS‐200, Schiller, Baar, Switzerland). Prior to each test we inserted a catheter (BD Arterial Cannula 20G 1.1 × 45 mm, Franklin Lakes, NJ) into the radial artery for blood sampling.

The participants performed two maximal incremental ramp tests (CPET_1_ and CPET_2_), 24 h apart, on a cycle ergometer (Ergometrics 900, Ergoline, Bitz, Germany). Gas exchange and ventilation were measured breath‐by‐breath (Oxycon Pro). The flow sensor was calibrated with a 3 L syringe prior to each test (Hans Rudolph, Shawnee, KS), and the gas analyzer for O_2_ and CO_2_ was calibrated against commercial standards. The increment rate was set individually, with the aim of reaching VO_2peak_ within 8 to 12 min of exercise. The rate was based on previous and current level of activity, physical examination, age, height and weight, and ranged from 10 to 24 W/min for the ME/CFS patients, and 15–30 W/min for the controls. The protocol included a 2‐min resting phase and 2 min of unloaded pedaling at a rate of 60–75 rpm, followed by a linear increase in power output until volitional exhaustion or until the participant was unable to maintain a cycling frequency above 45 rpm. They were given strong vocal encouragement during the tests. ECG was monitored continuously. Criteria to terminate the exercise test were any signs of distress, such as pallor or dizziness, chest pain, significant arrhythmias, or signs of ischemia on ECG. After reaching VO_2peak_, the participants continued to breathe through the facemask for a 3‐min recovery period.

### Analyses of biochemical markers

Arterial blood samples were collected at rest before each CPET and every 30th second during exercise. Test tubes contained an antiglycolytic agent to prevent continued glycolysis in the blood samples (2.0 mL sodium fluoride, potassium oxalate BD Vacutainer^®^ Plus, Franklin Lakes, NJ). The samples were immediately placed in iced water and centrifuged at 1500*g* for 15 min at 21°C within 30 min (Sigma 2‐7, Osterode am Harz, Germany). Plasma was separated and put on dry ice before storing at −80°C until analyzed. Arterial lactate concentration ([La_a_]) was determined by spectrophotometer (MaxMat PL, Montpellier, France). We added 4‐chlorophenol, lactate oxidase, peroxidase and 4‐aminophenazone (Lactate, Spinreact, Girona, Spain) to plasma and the tubes were mixed at 37°C for 10 min. The absorbance of samples and standards was read at 505 nm against the blank, with a detection limit of 0.044 mmol/L and a linearity limit of 16.85 mmol/L. Control sera (SPINTROL H Normal and Pathologic, Spinreact, Girona, Spain) were used to monitor the performance of assay procedures, as recommended by the manufacturer. Arterial blood samples for analyses of hemoglobin concentration were collected at rest before each CPET and within 90 sec after peak exercise had been achieved, and analyzed within 1 minute after sampling (ABL835 version 6.16 Flex; Radiometer, Copenhagen, Denmark).

### Analyses of exercise variables

Variables measured breath‐by‐breath were averaged over a 30‐sec sampling time. The gas exchange threshold was visually identified by the V‐slope method (Schneider et al. 1993), independently by two investigators. Relative exercise intensity was defined as per cent of VO_2peak_ (%VO_2peak_). The point where [La_a_] started to accumulate was defined as the lactate turnpoint (LT) and was determined by log‐log transformation of [La_a_] versus power output, that is, at the intersection of their regression lines, as described by Beaver et al. (1985), and using a computer script designed for this purpose. Power output with its linear increase during test, was preferred above oxygen uptake in order to avoid the need of smoothing the oxygen curves prior to log‐log transformation, given that oxygen uptake not necessarily increases in a strictly linear fashion (Beaver et al. 1985; Goodwin et al. 2007). We also estimated power output for a fixed [La_a_] of 4 mmol/L, often referred to as the onset of blood lactate accumulation (OBLA) (Goodwin et al. 2007). A fitted line for [La_a_] during each individual CPET was visually inspected to find the corresponding power output (to the nearest 0.5 W).

### Sample size

According to a reproducibility study on CPET, a difference in VO_2peak_ of 10% represents a real change in exercise tolerance (Hansen et al. 2004). In a previous study on repeated CPET in subjects with ME/CFS, mean (SD) VO_2peak_ decreased with 6 (7.5) % from CPET_1_ to CPET_2_, corresponding to −1.33 (1.68) mL/kg/min (Vermeulen et al. 2010). To demonstrate a difference of 10% between the two tests, corresponding to 2.23 mL/kg/min, with a two‐sided significance level of 5% and 80% power; our study would require a sample size of 18 participants in the ME/CSF group.

### Statistics

Data are presented as mean (SD). Two‐sample and paired *t*‐tests were used for all CPET variables. Due to somewhat skewed distributions, [La_a_] at rest, at gas exchange threshold and at LT were also analyzed after log_10_ transformation, but this did not change the results (data not shown). Mixed model analysis for repeated measurements with random intercept and random effect of power output was applied for [La_a_] during the tests. Both power output and the square of power output were included in the model. Group and day were modeled as fixed effects. We tested for interaction between group and test, and between group and power output and the square of power output (i.e. whether there were group differences in [La_a_] accumulation with increasing power output). To detect whether the difference in [La_a_] curves from CPET_1_ to CPET_2_ was significantly different between the groups, we calculated the change in [La_a_] for each power output per kilo body weight (PO_kg_) in every participant. We performed mixed model analyses on the calculated change in [La_a_], with fixed intercept, random effect of PO_kg_, and with group modeled as fixed effect, to test for interaction between group and PO_kg_. *P*‐values less than 0.05 were considered statistically significant. Statistical analyses were performed with SPSS (IBM SPSS Statistics version 22, Armonk, NY).

## Results

### Subject characteristics

**Figure 1:**
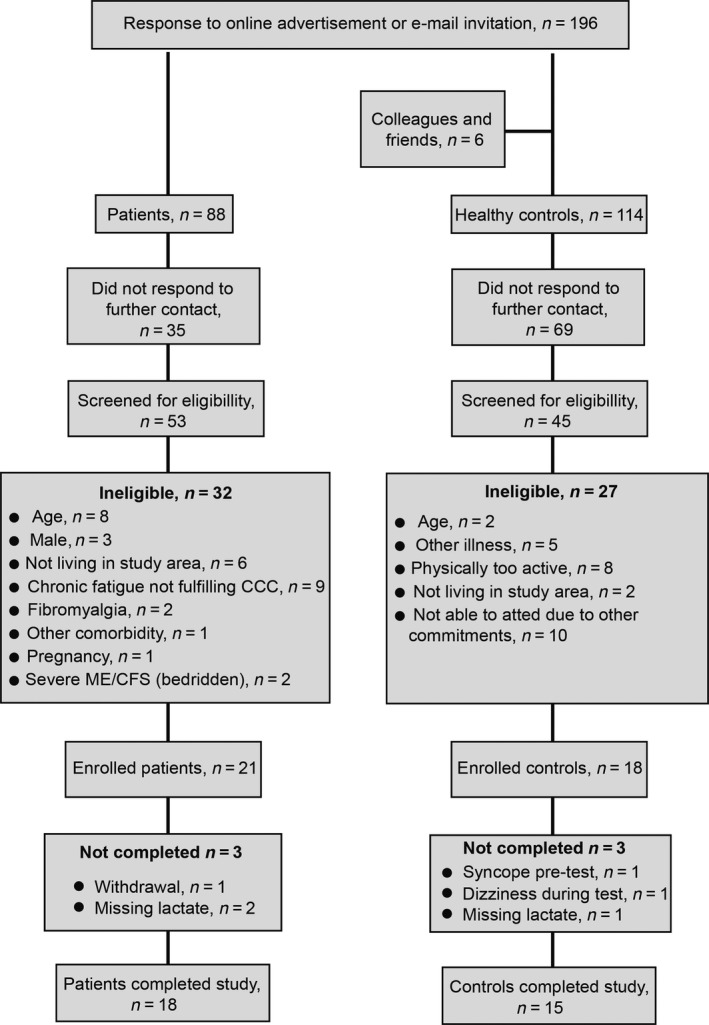
Flowchart of the inclusion of participants.

The inclusion process is illustrated in Figure 1, and the subject characteristics of the 18 patients and 15 controls that completed both CPETs with arterial plasma samples for lactate analyses are reported in Table 1. Age and height were similar in the two study groups, but the patients had higher body weight and BMI than the controls. Spirometry prior to CPET was within normal limits in both groups and did not indicate any obstructive or restrictive lung disorders. The hemoglobin concentration was also similar between the groups at CPET_1_ and CPET_2_.

**Table 1:** Baseline characteristics of the two study groups

Characteristic	ME/CFS (*n* = 18)	Controls (*n* = 15)	*P*‐value
Age (years; range)	38 (21–49)	34 (21–49)	0.14
Height (m)	1.71 (0.08)	1.69 (0.07)	0.46
Weight (kg)	73 (13)	63 (11)	0.02
BMI (kg/m^2^)	25.2 (5.0)	22.0 (3.2)	0.03
FVC (L)	4.43 (0.57)	4.41 (0.66)	0.95
FEV1 (L)	3.51 (0.46)	3.55 (0.56)	0.83
FEV1/FVC (%)	79.4 (5.0)	80.5 (6.0)	0.56
Predicted FVC (%)	121 (17)	121 (16)	0.97
Predicted FEV1 (%)	111 (17)	112 (15)	0.88
Hb day 1 (g/dL)	13.2 (0.9)	12.7 (0.8)	0.19
Hb day 2 (g/dL)	12.5 (0.9)	12.0 (0.8)	0.18

Values are mean (SD) unless otherwise reported.

### Peak exercise responses

**Figure 2:**
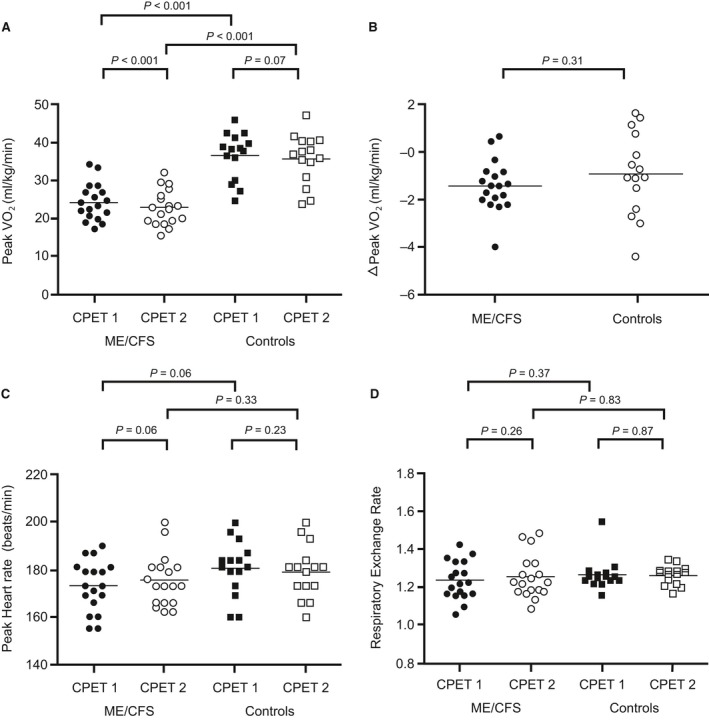
Peak exercise responses. Data points represent individual measures for each participant and horizontal lines are mean values. (A) shows VO_2peak_ for both CPETs and both study groups, while (B) shows the difference (Δ) in VO_2peak_ between CPET_1_ and CPET_2_ for both study groups. (C) shows peak heart rate for both CPETs and for both study groups, and (D) shows respiratory exchange rate at peak exercise for both CPETs and for both study groups.

**Figure 3:**
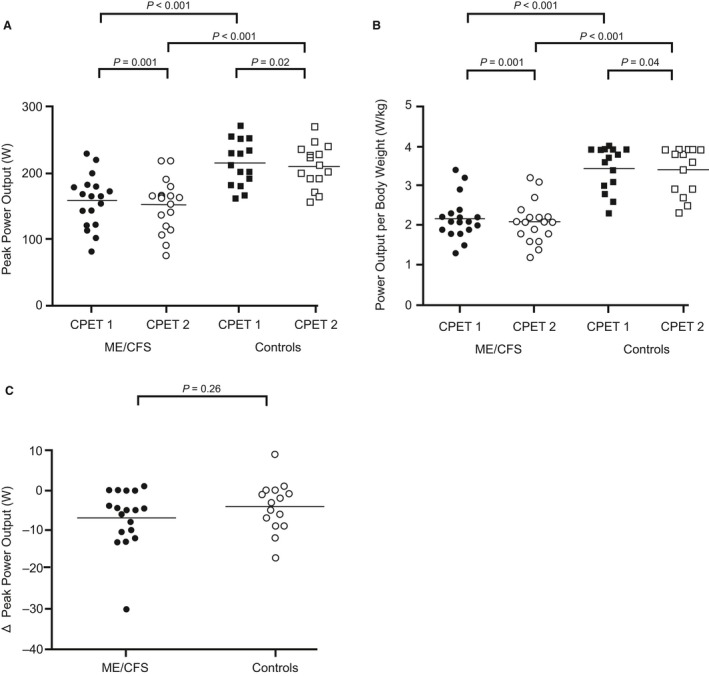
Power output at peak exercise. Data points represent individual measures for each participant and horizontal lines are mean values. (A) shows peak power output for both CPET_1_ and CPET_2_ and for both study groups, (B) shows peak power output adjusted for body weight, and (C) shows the difference (Δ) in peak power output between CPET_1_ and CPET_2_ for both study groups.

VO_2peak_ was significantly lower in patients than controls on both CPETs (Fig. 2A). VO_2peak_ was further reduced from CPET_1_ to CPET_2_ in patients, but the difference in peak VO_2peak_ between CPETs did not differ significantly between the two groups (Fig. 2B). Maximum heart rate and respiratory exchange rate did not differ significantly between the groups or between the two CPETs (Fig. 2C and D). Power output at peak exercise (PO_peak_) was significantly lower in patients than controls on both CPETs (Fig. 3A), also when analyzed for power output per body weight (PO_kg_) (Fig. 3B). Both groups had a significant reduction in PO_peak_ from CPET_1_ to CPET_2_. The difference in PO_peak_ between the two CPETs was not significantly different between the groups (Fig. 3C).

### Lactate concentrations at baseline and during tests

**Figure 4:**
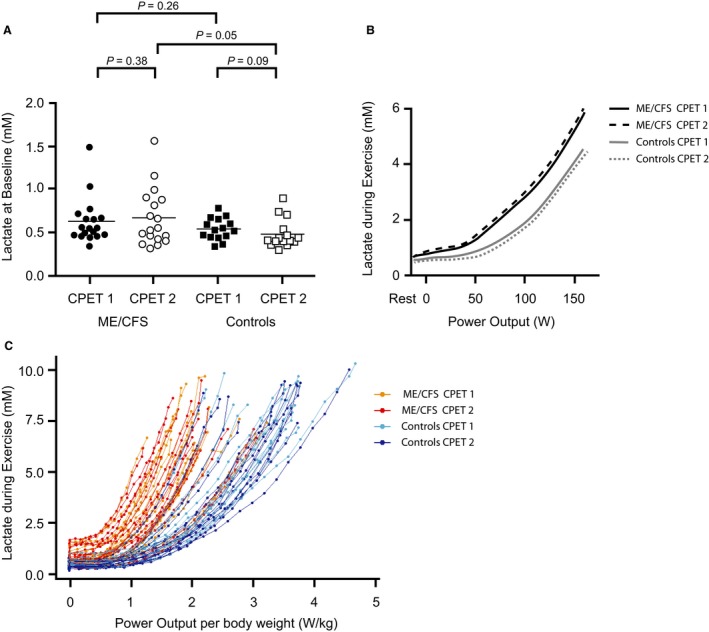
[La_a_] at baseline and during exercise. Data points represent individual measures for each participant and horizontal lines are mean values in (A), and shows resting [La_a_] at baseline prior to each CPET for both study groups. (B) shows mean [La_a_] curves for both study groups on both days up to 150 W, as this was the mean peak power output in the patient group. (C) shows the raw data of all [La_a_] samples and data point represents individual [La_a_] measures for each participant per power output per body weight. Lines represent individual curves of [La_a_] per power output per body weight.

Resting [La_a_] did not differ between patients and controls before CPET_1_, but were significantly different before CPET_2_ (Fig. 4A). In the mixed model analyses, [La_a_] per power output and PO_kg_ were significantly higher in patients than in controls on both CPETs (*P*_interaction_ < 0.001; Fig. 4B and C). Furthermore, the difference in [La_a_] per power output and PO_kg_ between the groups increased from CPET_1_ to CPET_2_ (*P*_interaction_ < 0.001; Fig. 4B and C). The [La_a_] curve for CPET_2_ was shifted significantly to the left in patients (*P* < 0.001) and to the right in controls (*P* < 0.001), compared to the [La_a_] curves for CPET_1_ (Fig. 4B and C).

### Gas exchange threshold

**Figure 5:**
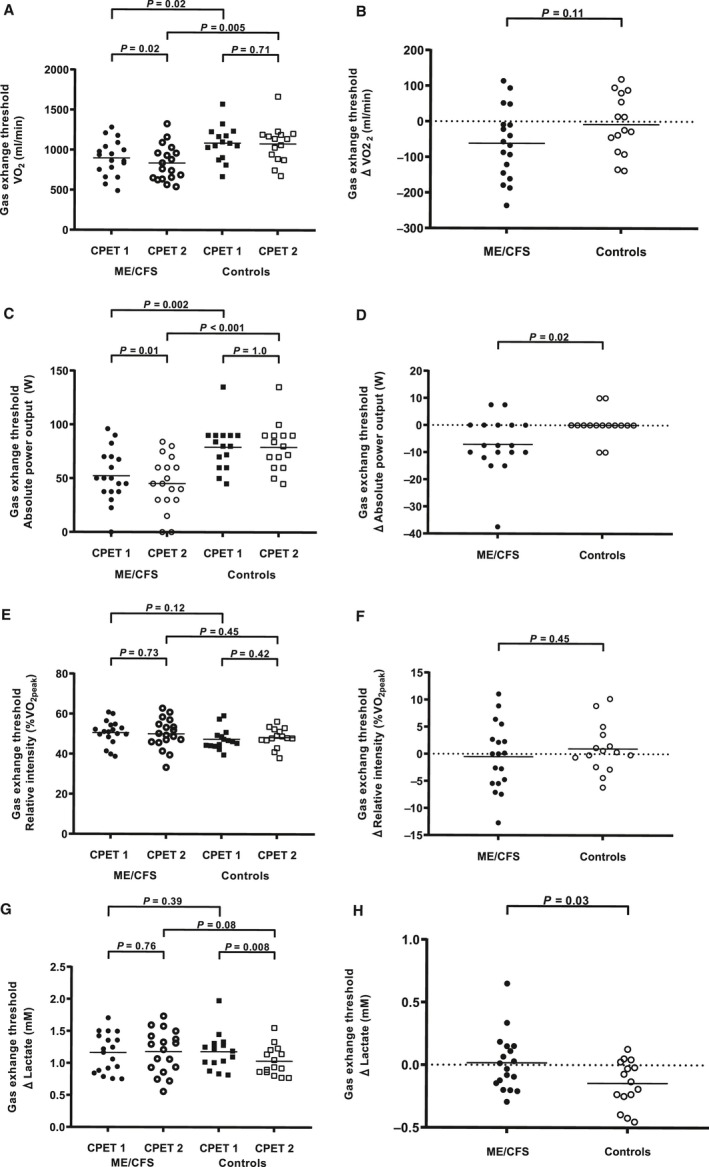
Exercise responses and [La_a_] at the gas exchange threshold (GET) defined by the V‐slope method. Data points represent individual measures for each participant and horizontal lines are mean values. (A) shows VO_2_ at GET for both CPETs and both study groups. (B) shows the difference (Δ) in VO_2_ at GET between CPET_1_ and CPET_2_ for both study groups. (C) shows the absolute power output at GET for both CPETs and both study groups. (D) shows the difference (Δ) in absolute power output at GET between CPET_1_ and CPET_2_ for both study groups. (E) shows the relative exercise intensity as %VO_2peak_ at GET for both CPETs and both study groups. (F) shows the difference (Δ) in %VO_2peak_ at GET between CPET_1_ and CPET_2_ for both study groups. (G) shows [La_a_] at GET for both CPETs and both study groups. H shows the difference (Δ) in [La_a_] at GET between CPET_1_ and CPET_2_ for both study groups.

VO_2_ at gas exchange threshold (GET) was significantly lower in patients than controls on both CPETs (Fig. 5A). VO_2_ at GET was further reduced in patients from CPET_1_ to CPET_2_, but the difference in VO_2_ at GET did not differ significantly between groups (Fig. 5B). The power output at GET was significantly lower in patients compared to controls on both CPETs, and was further reduced on CPET_2_ (Fig. 5C). The difference in power output at GET was significantly different between groups (Fig. 5D). The relative exercise intensity (%VO_2peak_) did not differ significantly between patients and controls on either CPET (Fig. 5E and F). The [La_a_] at GET was not significantly different between groups on CPET_1_, but was significantly reduced in controls on CPET_2_ (Fig. 5G), and the difference in [La_a_] at GET from CPET_1_ to CPET_2_ was significantly different between patients and controls (Fig 5H). Neither the respiratory exchange rate nor the heart rate at GET differed significantly between groups or the CPETs (data not shown).

### The lactate turnpoint and onset of blood lactate accumulation

**Figure 6:**
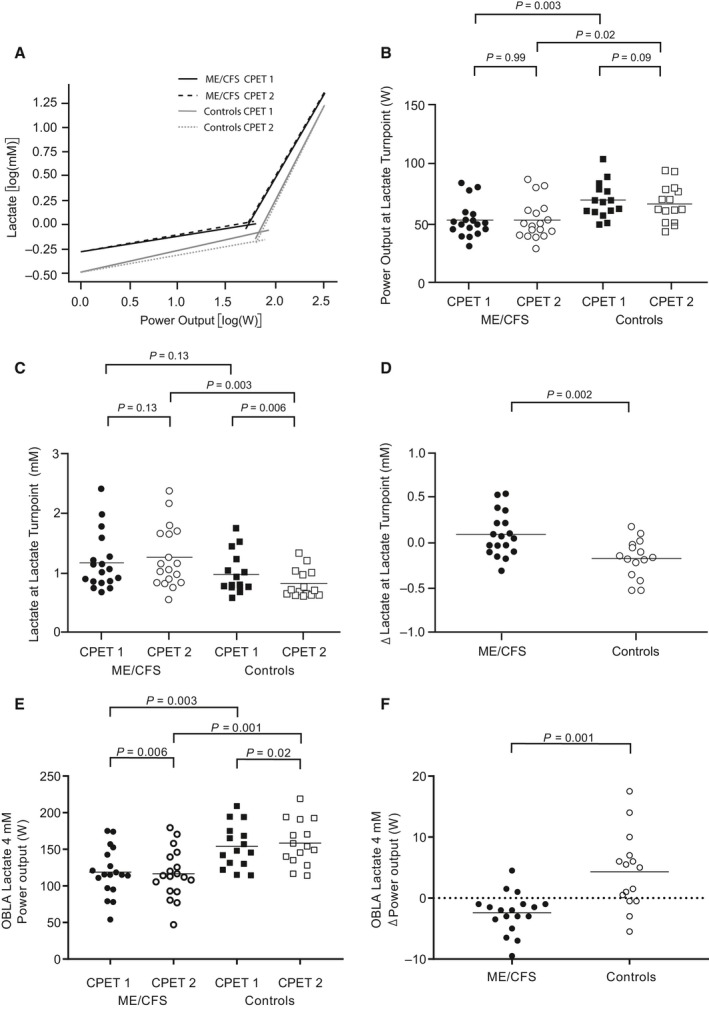
Power output and [La_a_] at the lactate turnpoint (LT) and at the onset of blood lactate accumulation of 4 mmol/L (OBLA). (A) shows the determination of LT by log‐log transformation of [La_a_] versus power output for both CPETs and both study groups. The intersection of the two regression lines defines the LT. Data points in (B) to (F) represent individual measures for each participant and horizontal lines are mean values. (B) shows the power output at LT for both CPETs and both study groups. (C) shows the [La_a_] at LT for both CPETs and both study groups, while (D) shows the difference (Δ) in [La_a_] between CPET_1_ and CPET_2_ for both study groups. (E) shows the power output at OBLA for both CPETs and both study groups, and (F) shows the difference (Δ) in power output at OBLA between CPET_1_ and CPET_2_ for both study groups.

The lactate turnpoint (LT) occurred at a significantly lower power output in patients on both CPETs, but neither group had any significant difference in power output between CPETs (Fig 6A and B). The [La_a_] at LT was not significantly different between groups on CPET_1_, but was significantly reduced in controls from CPET_1_ to CPET_2_ (Fig. 6C). The difference in [La_a_] at LT between CPETs was significantly different between the groups (Fig. 6D). The onset of blood lactate accumulation (OBLA) occurred at a significantly lower power output in patients compared to controls on both CPETs. The power output at OBLA increased significantly in controls and decreased significantly in patients from CPET_1_ to CPET_2_ (Fig. 6E), and the difference in power output at OBLA from CPET_1_ to CPET_2_ was significantly different between groups (Fig. 6F).

## Discussion

In this study of repeated CPET in ME/CFS patients and healthy controls, VO_2peak_ and VO_2_ at GET decreased significantly in patients, but not in controls, from CPET_1_ to CPET_2_, but the differences in VO_2peak_ and VO_2_ at GET between the two CPETs did not differ significantly between the two study groups. However, the patients had elevated [La_a_] for any absolute power output compared with the healthy controls, and both GET and LT occurred at a significantly lower VO_2_ and absolute power output in patients. Both study groups had similar relative exercise intensity (%VO_2peak_) at GET on both CPETs. When retested after 24 h, controls had no significant reduction in the power output at GET*,* but the [La_a_] at GET was significantly reduced. In contrast, patients had a significant reduction in the power output at GET on CPET_2_, while no significant reduction in [La_a_] at GET. Power output at OBLA demonstrated the same pattern, with a decrease in patients and an increase in controls from CPET_1_ to CPET_2_. Pre‐exercise [La_a_] was also significantly different between the two study groups before CPET_2_. Whereas previous exercise (CPET_1_) led to lower [La_a_] per absolute power output (right curve shift) on CPET_2_ in controls, [La_a_] per absolute power output increased and the curve was shifted to the left on CPET_2_ in patients.

Repeated measurements of cardiopulmonary exercise test variables have generally shown good reproducibility in patients with moderate exercise impairment due to chronic disease, and can also be reliably and reproducibly assessed in patients with severe exercise intolerance (Hansen et al. 2004). Although our patients had a reduced activity level compared to their pre‐illness capacity, none of them were bedridden or severely physically restricted. All participants had normal hemoglobin concentration, normal spirometry values prior to the CPETs, as well as a normal to high breathing reserve at peak exercise. Blood sampling during CPET_1_ amounted to approximately 150–200 mL blood which could explain the reduction in resting hemoglobin concentrations between the two CPETs (Schaffartzik et al. 1993). Mean respiratory exchange ratio and maximum heart rate were similar in both groups on both CPETs, and the findings are therefore not likely explained by deliberate underperformance or lack of effort. Patients and healthy controls were similar in gender, age and height, but patients were heavier than controls. We therefore also reported results for power output per body weight, but this did not affect the results.

As opposed to what others have found, the ability to reproduce VO_2peak_ did not differ significantly between the two study groups. Although VO_2peak_ decreased significantly in patients, the absolute change was too small, in our opinion, to indicate any major change in exercise tolerance or explain PEM. Furthermore, the decrease in resting hemoglobin concentration of 0.7 g/dL in both study groups from CPET_1_ to CPET_2_ should be taken into consideration. As an average, a reduction in hemoglobin concentration of 1 g/dL accounts for a reduction in peak VO_2_ of 0.97 mL/kg/min (Agostoni et al. 2010). In our study, this would equal an expected mean reduction in peak VO_2_ of 0.68 mL/kg/min from CPET_1_ to CPET_2_.

The test protocol did show differences in [La_a_] between ME/CFS patients and healthy controls on both CPETs, and this difference increased on CPET_2_. Untrained subjects will rapidly improve their lactate clearance ability if they engage in regular endurance training (Donovan and Brooks 1983; Phillips et al. 1995), thus lowering blood lactate concentrations for a given work load (Holloszy and Coyle 1984; Yoshida et al. 1992). Such a right shift of the lactate curve can be demonstrated as early as 24 h after prior exercise (Neary and Wenger 1985), and fits well with the findings in our control group on CPET_2_. [La_a_] per absolute power output is elevated in untrained compared to trained subjects (MacRae et al. 1992), and can explain the difference in [La_a_] between patients and controls on CPET_1_. However, one would still have expected a right shift on the second day in patients as well; similar to what was observed in the controls.

GET occurred at a similar relative exercise intensity defined as % of VO_2peak_ for each CPET in both patients and controls. On CPET_1_ patients had a lower power output at GET than controls, but [La_a_] was similar between the groups. On CPET_2_ patients had a reduction in power output at GET*,* but similar [La_a_], whereas controls had similar power output at GET*,* but reduced [La_a_]. This inability to reproduce power output at GET seems to be a consistent finding in patients with CFS/ME (VanNess et al. 2010; Vermeulen et al. 2010; Snell et al. 2013; Keller et al. 2014; Hodges et al. 2018). If this was caused by deconditioning alone, one would expect to find similar results in other conditions where deconditioning, low exercise tolerance and fatigue are prevalent. However, patients with sarcoidosis (Braam et al. 2013) and multiple sclerosis (Hodges et al. 2018) are able to reproduce their power output at GET, despite a low exercise capacity.

LT occurred at a lower absolute power output in patients than in controls. The power output at LT did not differ between the two CPETs in either group, contrary to our findings when we applied the V‐slope method. One explanation might be that some patients probably reached their LT during unloaded pedaling on CPET_2_, at which point we did not have lactate measures every 30th second. This would affect the computed regression lines. Nonetheless, [La_a_] showed the same pattern, with similar [La_a_] on CPET_1_ and significant differences in [La_a_] between the two study groups on CPET_2_.

A left shift of the lactate curve in ME/CFS patients was suggested by Lane et al. (1994), but subsequent studies have not been conclusive. For example, maximal exercise testing of healthy subjects and ME/CFS patients diagnosed according to the Fukuda criteria did not show any abnormalities in maximal oxygen uptake, lactate accumulation or differences in LT determined by the log‐log method (Sargent et al. 2002), or in venous lactate versus VO_2_ (Jammes et al. 2005). Others have found elevated lactate accumulation on submaximal levels of exertion in patients diagnosed according to the CDC 1988 working case definition (Holmes et al. 1988; Riley et al. 1990), as well as in a proportion of ME/CFS patients fulfilling the rather unspecific Oxford criteria, where subjects with normal lactate concentrations were more likely to suffer from psychiatric comorbidities (Lane et al. 1995, 1998). Two other studies did not find any differences in resting or maximal lactate concentrations, but found significantly lower postexercise lactate concentrations in patients (Gibson et al. 1993; Georgiades et al. 2003). The discrepancies may be explained by different diagnostic criteria and different exercise protocols. To our knowledge, repeated exercise testing with lactate profiles on two consecutive days has not been studied in ME/CFS patients fulfilling case definitions where PEM is a mandatory symptom.

LT seems to correspond to a limitation in the metabolic clearance rate where lactate appearance exceeds lactate disposal, regardless of training status, and lactate appearance seems to be closely related to %VO_2peak_. Furthermore, blood lactate concentrations at same relative intensity are similar in trained and untrained (Messonnier et al. 2013). Training increases intramuscular lactate clearance primarily by increasing oxidation by upregulation of mitochondrial proteins, and reduces net lactate production in muscle due to facilitated lactate exchange between glycolytic and oxidative fibers. Patients and controls had similar [La_a_] at similar relative exercise intensity on CPET_1_, but controls had a reduction in [La_a_] at both relative and absolute intensity on CPET_2_ that was not seen in patients. We propose that the previous exercise led to an improved lactate disposal in controls on CPET_2_, as their [La_a_] was reduced both at rest, at the gas exchange threshold and at the lactate turnpoint, and as the onset of blood lactate accumulation at 4 mmol/L occurred at a higher absolute power output.

Disturbed energy metabolism in ME/CFS has been demonstrated by several investigators. Myoblasts grown in presence of serum from severe ME/CFS patients show increased mitochondrial respiration and increased lactate secretion (Fluge et al. 2016). Peripheral blood mononuclear cells (PBMC) from ME/CFS patients have an impaired maximal respiration capacity compared to healthy PBMCs, suggesting an inability to adequately increase the respiratory rate in response to elevated metabolic stress (Tomas et al. 2017). In vitro electric pulse stimulation of muscle cells as a model to investigate metabolic changes during exercise has shown impaired AMPK phosphorylation and glucose uptake in cells from ME/CFS patients and diminished release of IL‐6 compared to healthy muscle cells (Brown et al. 2015). Exercise leads to a transient upregulation of pyruvate dehydrogenase kinase (PDK), particularly during recovery, but returns to resting values within 24 h (Pilegaard and Neufer 2004). PBMCs from ME/CFS patients show upregulated expression of PDK (Fluge et al. 2016), proposing a disturbed PDK regulation which could limit the pyruvate flux with the potential to affect the clearance of lactate through oxidation. Patients with ME/CFS have elevated lipopolysaccharide (LPS) levels in blood compared to healthy controls (Giloteaux et al. 2016). Exercise leads to increased bacterial translocation in ME/CFS patients and is proposed as a possible cause for PEM (Shukla et al. 2015). LPS could affect metabolism, either through inflammation and/or elevated catecholamines. Several conditions are associated with elevated lactate, and elevated lactate might be an attempt to mitigate the effects of injury and stress rather than causing it (Brooks 2018).

This study was performed on female patients with mild to moderate degree of ME/CFS. Diagnosing patients based on clinical criteria rather than a valid biomarker carries the risk of including a less representative group. However, no such biomarker exists today. Ideally one should have patients and controls with similar activity level, as well as similar body weight. The strengths of this study are that patients fulfilled the Canadian criteria with PEM as a required symptom, and all patients were evaluated by the same physician prior to inclusion. All included participants reached predefined test criteria for maximal exercise testing and arterial lactate was sampled every 30th second. All tests were supervised by the same staff, and the testing conditions were similar for all participants, including time of day, metabolic state prior to tests (fasting), and that most patients stayed at the test facility in order to eliminate additional exertion between the tests. It should be noted that the patients experienced considerable symptom exacerbation after the tests, often lasting for weeks.

Exercise intolerance, PEM and delayed recovery are prominent symptoms in ME/CFS. Intriguingly, this study indicates that previous exercise increases lactate accumulation in ME/CFS as opposed to the reduction seen in healthy controls, although the mechanism for this has yet to be established. We do not know whether this finding points to a central pathophysiological mechanism in ME/CFS, or if it is a secondary phenomenon. It might even be an attempt to alleviate the negative impact that exercise seems to have in these patients. Further research is needed in order to elucidate the causes of this abnormal response to exertion.

## Conflict of Interest

None declared.
